# Automated AI-based Mayo Endoscopic Scoring for ulcerative colitis across adult and pediatric cohorts from diverse populations

**DOI:** 10.1093/ecco-jcc/jjag122

**Published:** 2026-08-18

**Authors:** Kamal Hammouda, Rishi Dakarapu, Chathruckan Rajendra, Hyojeong Lee, Sahar Almahfouz Nasser, Sharmistha Rudra, Vasantha Kolachala, Caitlin Diefendorf, Irina Geiculescu, Sushma Maddipatla, Anant Madabhushi, Subra Kugathasan

**Affiliations:** Wallace H. Coulter Department of Biomedical Engineering, Emory University and Georgia Institute of Technology, Atlanta, GA, 30322, United States; Atlanta Veterans Affairs Medical Center, Decatur, GA, 30033, United States; Mathematics Department, Faculty of Science, Mansoura University, Mansoura, 35516, Egypt; Wallace H. Coulter Department of Biomedical Engineering, Emory University and Georgia Institute of Technology, Atlanta, GA, 30322, United States; Department of Pediatrics & Pediatric Research Institute, Emory University, Atlanta, GA, 30322, United States; Wallace H. Coulter Department of Biomedical Engineering, Emory University and Georgia Institute of Technology, Atlanta, GA, 30322, United States; Health Screening and Promotion Center, Asan Medical Center, University of Ulsan College of Medicine, Seoul, 05505, Republic of Korea; Wallace H. Coulter Department of Biomedical Engineering, Emory University and Georgia Institute of Technology, Atlanta, GA, 30322, United States; Department of Pediatrics & Pediatric Research Institute, Emory University, Atlanta, GA, 30322, United States; Department of Pediatrics & Pediatric Research Institute, Emory University, Atlanta, GA, 30322, United States; Department of Pediatrics & Pediatric Research Institute, Emory University, Atlanta, GA, 30322, United States; Department of Pediatrics & Pediatric Research Institute, Emory University, Atlanta, GA, 30322, United States; Department of Pediatrics & Pediatric Research Institute, Emory University, Atlanta, GA, 30322, United States; Wallace H. Coulter Department of Biomedical Engineering, Emory University and Georgia Institute of Technology, Atlanta, GA, 30322, United States; Atlanta Veterans Affairs Medical Center, Decatur, GA, 30033, United States; Department of Pediatrics & Pediatric Research Institute, Emory University, Atlanta, GA, 30322, United States

**Keywords:** ulcerative colitis (UC), Mayo Endoscopic Score (MES), deep learning, domain shift, pediatric and adult

## Abstract

**Background:**

Endoscopic assessment in ulcerative colitis (UC) is critical for clinical decision-making but limited by interobserver variability. AI-powered systems may improve consistency, but dataset heterogeneity has hindered clinical translation. We developed and evaluated a deep learning framework for standardized Mayo Endoscopic Score (MES) prediction across adult and pediatric populations from diverse regions.

**Patients and methods:**

Using three multi-institutional datasets, we trained and validated convolutional neural network models to predict MES. The primary dataset (LIMUC; 564 adults, 11 276 images, Turkey) supported model development, with external validation on TMC (308 adults, 7978 images, China) and the Emory Pediatric dataset (EPC; 80 children, 113 images, USA). Two models were developed: UC-Re for binary remission classification (MES 0-1 vs 2-3) and UC-MES for four-class grading (MES 0-3). Imaging artifacts were corrected using inpainting, and Fourier-Spatial Image Harmonization (FSIH) mitigated inter-institutional domain shifts. Performance was evaluated using area under the operating characteristic curve (AUC), F1-score, and quadratic weighted kappa (QWK).

**Results:**

UC-Re achieved AUCs of 0.98, 0.95, and 0.98 across LIMUC, TMC, and EPC, with F1-scores of 0.92, 0.87, and 0.93, respectively. UC-MES demonstrated strong ordinal consistency (QWK = 0.81-0.85), comparable to inter-expert agreement (QWK = 0.88). Most misclassifications occurred between adjacent MES categories, reflecting human-like patterns.

**Conclusions:**

This novel AI-based framework predicts MES across geographically diverse adult and pediatric UC datasets. Its strong performance, including comparability to expert gastroenterologists in EPC, supports its potential as a decision-support tool for standardized endoscopic monitoring. However, prospective multicenter validation is warranted prior to routine clinical implementation.

## 1. Introduction

Ulcerative Colitis (UC) is a chronic inflammatory bowel disease characterized by continuous inflammation and ulceration of the colonic mucosa, typically beginning in the rectum and extending proximally.^1^ The global epidemiology of UC exhibits substantial geographic variation: the United States reports one of the highest incidence rates at approximately 32.5 cases per 100 000 persons.^2^ Regions with historically lower incidence, such as parts of Asia and South America, are experiencing rising rates.^2^^,^^3^ In many regions, prevalence is rising steeply among children and adolescents, with the age of diagnosis reducing. Pediatric UC often presents with more extensive colitis at diagnosis compared with adult-onset disease.^1^^,^^2^

Endoscopic evaluation remains the gold standard for assessing UC disease activity, providing direct visualization of the mucosa, and is recommended by American and British clinical guidelines for staging and therapeutic decision-making.^3^^,^^4^ Mucosal healing has been shown to correlate strongly with long-term outcomes such as reduced colectomy risk and sustained remission.^5^ The Mayo Endoscopic Score (MES), ranging from 0 to 3, is a widely used index to grade mucosal disease severity in trials and clinical practice, with scores of 0-1 indicating remission and 2-3 reflecting active disease.^6^ However, the clinical utility of MES is limited by substantial inter- and intra-observer variability.^7^ Although alternative scoring systems and structured training programs have been attempted, they have demonstrated limited scalability and only modest improvements in reproducibility.^8^

Artificial intelligence (AI), particularly deep learning (DL), offers a promising alternative.^9^^,^^10^ By leveraging large, annotated datasets, DL models can automate MES grading, potentially reducing subjectivity and enhancing diagnostic consistency.^11^ A range of convolutional neural network (CNN) architectures has achieved performance levels comparable to human experts in adult UC datasets.^12^^,^^13^ AI tools have also been tested in real-time settings and have shown an ability to assist non-inflammatory bowel disease (IBD) experts and improve reproducibility in workflow settings.^14^^,^^15^ However, challenges remain around model interpretability, which is critical for clinical adoption. Generalizability is also particularly difficult due to reliance on limited homogeneous datasets (in terms of geography, patient population, and imaging equipment), as models developed could potentially perform differently in underrepresented populations in clinical trials.^16^^,^^17^ In addition, existing methods may not sufficiently address imaging artifacts, lighting differences, biological differences, and other cross-institutional domain shifts.

Another important gap is the limited evaluation of pediatric populations in AI-based endoscopic assessment of UC.^18^ Most existing DL models have been developed exclusively on adult UC datasets,^11^^,^^19^ and their generalizability to pediatric populations has not been adequately established. Given that pediatric UC represents a clinically distinct population in terms of disease presentation and extent, evaluating whether models can generalize to pediatric cohorts without retraining constitutes an important unmet need in pediatric endoscopy AI.

In this study, using multi-institutional endoscopic image datasets (from Asia, the Middle East, and North America), we developed and validated an automated DL pipeline to assess MES in both pediatric and adult UC patients. A key innovation is the use of Fourier-Spatial Image Harmonization (FSIH), designed to mitigate domain shifts across institutions and imaging conditions. Combined with artifact removal via artificial inpainting, our pipeline aims to deliver high reproducibility and generalizability. We trained and validated CNN models (eg, ResNet50) for four-class MES prediction and binary remission classification, achieving high accuracy across diverse datasets. This study presents a novel AI model evaluated across both pediatric and adult datasets from different geographic regions. We also sought to understand whether the performance of the AI tool was comparable to that of experienced human raters in the pediatric cohort.

## 2. Materials and methods

### 2.1. Dataset description and image labeling

**Figure 1.**
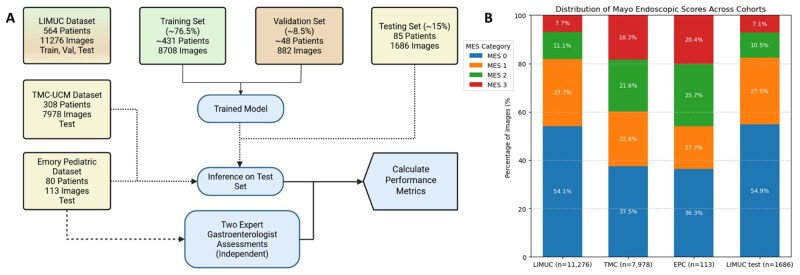
Study design and datasets. (A) The LIMUC dataset was split into training, validation, and testing sets for model development, with external validation on TMC-UCM and Emory pediatric datasets. Model predictions were compared against expert gastroenterologist assessments, and performance metrics were calculated. (B) Distribution of Mayo endoscopic scores (MES 0-3) across the LIMUC, TMC-UCM, and Emory pediatric datasets, as well as the LIMUC held-out test set.

This study utilizes three multi-institutional datasets to develop and validate DL models for assessing UC disease activity (Figure 1). The Labeled Images for Ulcerative Colitis (LIMUC), the primary dataset, is publicly available and contains 11 276 endoscopic images from 564 UC patients collected at the Marmara University Institute of Gastroenterology in Turkey.^19^ The dataset was used for training (∼76.5%), validation (∼8.5%), and internal testing (∼15%). Importantly, the train/validation/test split was performed at the patient level, ensuring full patient disjointness; no patient contributed images to more than one subset. The second dataset, Tongji Medical College (TMC), was exclusively used for external testing. It is a public dataset of 7978 endoscopic images from 308 UC patients from China.^20^ Available cohort-level clinical descriptors are summarized in Table S1. Age and sex distributions were available for LIMUC but not for TMC-UCM (Tongji Medical College–Ulcerative Colitis with Mayo Scores). Detailed patient-level clinical metadata were not uniformly available across datasets; however, both public cohorts were described as adult populations in the source datasets.

The third dataset, the Emory Pediatric Dataset (EPC), was also exclusively used for external validation. A total of 113 endoscopic images were obtained from 80 pediatric patients aged 18 years or less with UC receiving care at Children’s Healthcare of Atlanta (CHoA), United States. Images were collected from 2012 to 2024. The dataset had an average age at diagnosis of 11 years old. Of these, 51% identified as male at birth and 49% identified as female. The racial composition was distributed with 54% identifying as White, 8% as Asian, and 38% as Black/African American (Figure 1). This study protocol was approved by the Emory University Institutional Review Board [IRB00115045], and physicians were expected to have obtained informed consent prior to endoscopic procedures, as per the ethical protocols at CHoA facilities.

For the publicly available LIMUC and TMC datasets, we retained the original MES labels (0-3) to preserve consistency with the source datasets and enable comparison with published benchmarks. LIMUC used an independent blinded review by two experienced gastroenterologists, with discrepant cases adjudicated by a third reviewer and finalized by majority voting.^19^ TMC-UCM used review by at least three IBD-expert gastroenterologists, with disagreements resolved by consensus discussion and final review by an IBD specialist.^20^ For the EPC, MES assigned by the treating gastroenterologist at the time of diagnosis were used as the ground-truth labels, reflecting real-world clinical assessment. During this study, two board-certified gastroenterologists (R1 and R2) independently re-scored all EPC images using standard MES criteria, both blinded to the original diagnosis and to each other’s scores, serving as independent expert comparators.

### 2.2. Approach

A two-stage preprocessing pipeline was implemented to address imaging artifacts and mitigate domain shifts across datasets from different institutions. First, to correct for endoscopic artifacts such as specular glare, instrument shadows, and occlusions, we employed two complementary artificial inpainting models (AIMs): Contextual Artifact Inpainting (CAI) and the Large Mask Inpainting with Fourier Convolutions (LaMa). CAI was applied to repair small artifact regions, leveraging its strength in context-aware restoration of fine structures,^21^ while LaMa was used for larger missing or distorted areas due to its superior performance on extensive inpainting tasks.^22^^,^^23^ More details on this approach are provided in the Supplementary Methods. This strategy ensures optimal reconstruction of both localized and broad defects, preserving critical tissue and mucosal features (see Figure S1A).

#### 2.2.1. Fourier-spatial image harmonization

We implemented a two-stage normalization strategy, FSIH, to address domain discrepancies across datasets. This technique leverages histogram matching in both the frequency and spatial domains to align image characteristics while preserving anatomical content (Figure S1B). In the first stage, Fourier-domain histogram matching is performed across the entire magnitude spectrum of the image. Unlike earlier approaches such as Fourier Domain Adaptation (FDA),^24^^,^^25^ which modifies only the low-frequency components of the amplitude spectrum, our first stage matches the entire magnitude spectrum. In the second stage, spatial histogram matching is then applied to the pixel intensity distributions. This refines local details and addresses remaining discrepancies not addressed in the frequency domain, ensuring the final image closely matches the statistical characteristics of the target domain. Importantly, in both stages, all images, including those from the training, validation, and external test sets, were normalized to a single fixed reference image selected from the LIMUC training set. This fixed-reference normalization strategy ensures that no test image statistics are accessed at inference time, making the preprocessing pipeline fully inductive and deployment-ready. By anchoring all images to a consistent reference, FSIH effectively reduces inter-institutional domain shift while remaining applicable in prospective clinical settings without modification.

#### 2.2.2. Deep learning model implementation

**Figure 2.**
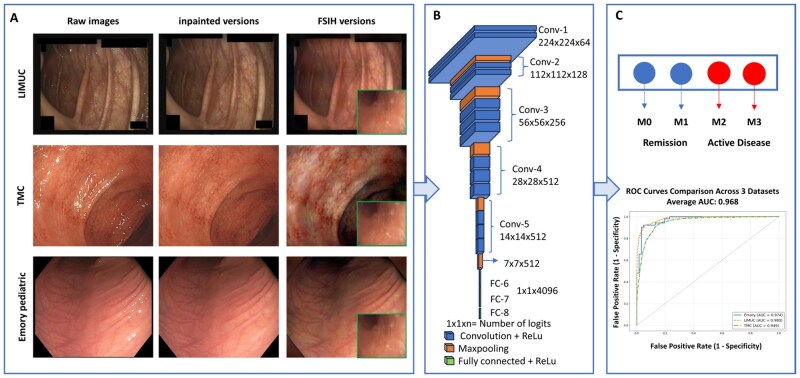
Overview of the proposed deep learning pipeline for Mayo Endoscopic Score (MES) classification. (A) Representative images from three datasets—LIMUC, TMC, and EPC—showing raw input, inpainted versions using contextual artifact inpainting (CAI), and domain-adapted versions using Fourier-Spatial Image Harmonization (FSIH). Insets highlight visual consistency after normalization. (B) ResNet50 architecture when input image size was 224 × 224. (C) MES classification scheme differentiating remission (MES 0-1) from active disease (MES 2-3). Receiver operating characteristic (ROC) curves across the three datasets show robust generalization.

The fully preprocessed images were then used as input to a ResNet50-based CNN for MES classification.^26^ We implemented two models: UC remission (UC-Re) (remission [0-1] vs active disease [2-3]) and UC-MES for four-class (0-3) prediction. A schematic overview of the complete preprocessing and classification workflow is shown in Figure 2. Full training details, including hyperparameters and data augmentation strategies, are included in the Supplementary Methods. To justify the selection of ResNet50, we systematically compared four CNN architectures, ResNet50, InceptionV3, DenseNet121, and EfficientNetB0, across both classification tasks. Processing times were measured on a Dell workstation (Intel Core i9-13950HX CPU) for preprocessing and a separate machine with a single NVIDIA RTX A5500 GPU for inference. The complete preprocessing pipeline (background cropping, specular-reflection removal, inpainting, and FSIH normalization) required approximately 188 ms per image, and model inference approximately 10 ms per image, for a total of about 198 ms per image end-to-end.

The LIMUC dataset was used for model development and initial internal testing. Trained models were then externally validated on the TMC and EPC datasets to assess generalizability. In addition to these primary tasks, two secondary experiments were conducted. First, a restricted binary task (MES 0 vs MES 3) was used to evaluate the model’s performance on clinically extreme cases. Next, to benchmark against expert interpretation, two gastroenterologists independently scored EPC images, and model predictions were compared with these labels using the evaluation metrics below.

#### 2.3. Statistical analysis

Model performance was evaluated using multiple complementary metrics. For the UC-Re model, we estimated accuracy, the area under the receiver operating characteristic (ROC) curve (AUC), F1-score, and Cohen’s kappa, with 95% confidence intervals (CIs). For the UC-MES model, quadratic weighted kappa (QWK) was used to account for the ordinal nature of the scores, along with accuracy and F1-score. To further examine robustness, performance was assessed separately on extreme cases only (MES 0 vs 3) using the same metrics. Comparisons between model predictions and expert gastroenterologists were performed using appropriate statistical tests for each metric: McNemar’s test for paired binary outcomes (accuracy), permutation testing for non-parametric F1-scores, Z-tests for kappa statistics, and bootstrapping for QWK. A two-sided *P*-value less than .05 was considered a statistically significant metric.^27^^,^^28^

For both the UC-Re and UC-MES models, image-level performance is the primary measure of model performance. All metrics are reported with 95% CIs estimated by patient-clustered bootstrap resampling (2000 resamples),^29^^,^^30^ in which patients rather than individual images were resampled to account for multiple images per patient. As a secondary analysis, performance was also evaluated at the patient level by aggregating each patient’s image predictions, using max-probability fusion for the binary UC-Re task and expected-grade fusion for the four-class UC-MES task. A patient was labeled active if at least one of their images was active; full details are provided in the Supplementary Methods.

## 3. Results

### 3.1. Study populations: adult and pediatric UC datasets

For performance evaluation, we analyzed 1686 images from the test subset of the LIMUC dataset, 7978 images from the TMC dataset, and 113 images from the EPC dataset. MES distributions differed substantially between datasets (Figure 1 and Table S1): LIMUC was dominated by remission (MES 0), and TMC contained a higher proportion of moderate-to-severe disease (active disease). At the same time, EPC was more evenly balanced across categories. The LIMUC dataset was stratified into training, validation, and internal testing using the publicly available standard split. Demographic information was available only for the EPC dataset, which included pediatric patients with a near-equal male-to-female distribution. These differences provided heterogeneous test conditions for model evaluation.

### 3.2. Detection of remission *vs* active disease (UC-Re model)

Across all three datasets, the UC-Re model demonstrated high accuracy and consistency in differentiating remission (MES 0-1) from active disease (MES 2-3). Using ResNet50 architecture with our preprocessing pipeline, the model achieved an F1-score = 0.92, accuracy = 0.95, Cohen’s Kappa = 0.84, and AUC = 0.98 on the LIMUC test set (*N* = 1686). Comparable performance was observed on the TMC dataset and on the EPC dataset (Figure S2A and Table 1). While ResNet50 consistently provided strong results across datasets, the other CNN-based architectures (Inception, DenseNet, and EfficientNet) also achieved comparable metrics across all datasets (Table 1).

**Table 1.** Performance metrics for two AI models: binary remission classification (UC-Re) and four-class MES classification (UC-MES) across three datasets (LIMUC, TMC-UCM, and Emory Pediatric dataset [EPC]).

Binary Remission Classification (UC-Re)												
	LIMUC (*N* = 1686)				TMC-UCM (*N* = 7978)				Emory Pediatric (*N* = 113)			
	F1	ACC	KAPPA	AUC	F1	ACC	KAPPA	AUC	F1	ACC	KAPPA	AUC
**Inception**	0.93	0.96	0.85	0.98	0.87	0.87	0.73	0.94	0.89	0.89	0.79	0.95
**DenseNet**	0.92	0.95	0.83	0.99	0.85	0.85	0.71	0.96	0.87	0.87	0.74	0.96
**EfficientNet**	0.91	0.95	0.83	0.99	0.86	0.86	0.72	0.95	0.90	0.90	0.80	0.96
**ResNet50**	**0.92**	**0.95**	**0.84**	**0.98**	**0.88**	**0.88**	**0.76**	**0.95**	**0.93**	**0.93**	**0.86**	**0.98**

Four-class MES Classification (UC-MES)									
	LIMUC (*N* = 1686)			TMC-UCM (*N* = 7978)			Emory Pediatric (*N* = 113)		
	F1	ACC	QWK	F1	ACC	QWK	F1	ACC	QWK
**Inception**	0.72	0.78	0.86	0.49	0.60	0.80	0.63	0.66	0.81
**DenseNet**	0.72	0.78	0.86	0.46	0.56	0.75	0.64	0.64	0.83
**EfficientNet**	0.72	0.77	0.86	0.52	0.62	0.82	0.59	0.61	0.80
**ResNet50**	**0.71**	**0.78**	**0.85**	**0.49**	**0.61**	**0.81**	**0.62**	**0.63**	**0.84**

**Figure 3.**
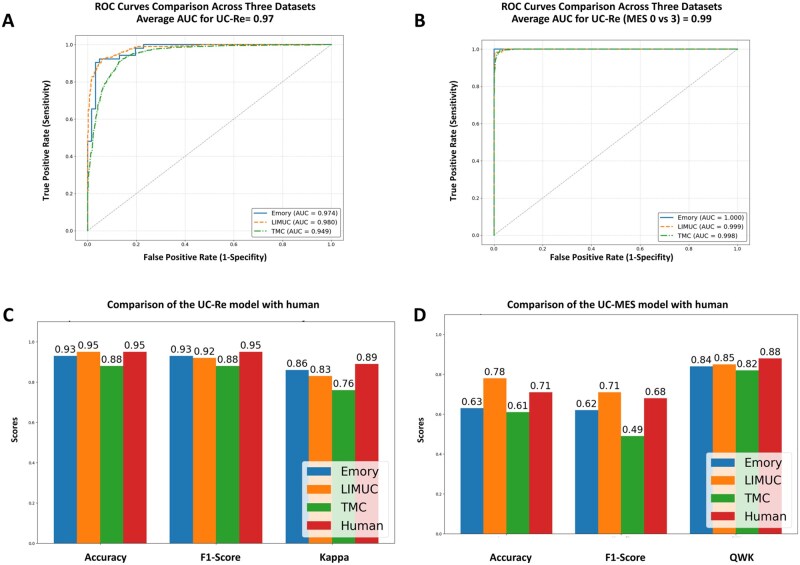
Model discrimination and comparison with expert reviewers across three cohorts. (A) ROC curves for the binary UC-Re task (active *vs* inactive disease) in the Emory, LIMUC, and TMC test sets (AUC 0.974, 0.980, and 0.949; average 0.97). (B) ROC curves for the MES 0 *vs* 3, shown as a separability check (AUC 1.000, 0.999, and 0.998). (C) UC-Re model compared with two board-certified gastroenterologists on the Emory cohort, measured by accuracy, F1-score, and Cohen’s κ. (D) UC-MES four-class model (MES 0-3) compared with the same reviewers, measured by accuracy, F1-score, and quadratic weighted κ (QWK). UC-Re, UC remission classification; UC-MES, Mayo Endoscopic Subscore grading; AUC, area under the ROC curve; QWK, quadratic weighted kappa.

The ROC analysis confirmed strong discrimination across datasets, with an average AUC of 0.97 (Figure 3A; Figure S2A). Analyzing the confusion matrices, in the test LIMUC dataset, 25/1389 (1.8%) of remission and 52/297 (17.5%) of active cases were misclassified. Similarly, for TMC: 728/4796 (15.2%) of remission and 255/3182 (8%) of active cases were misclassified. For EPC: 4/61 (6.6%) of remission and 4/52 (7.7%) of active cases were misclassified. The ResNet50 model achieved near-perfect separation between complete remission (MES 0) and severe disease (MES 3), misclassifying only 7/1045 (0.67%) cases in LIMUC and 96/4457 (2.15%) in TMC. There were zero misclassifications in the EPC dataset (0/64) (Figure 3B; Figure S2B). Preprocessing analysis showed that AIMs achieved the highest performance on the internal LIMUC dataset (F1 = 0.92, κ = 0.84), while FSIH demonstrated superior generalizability on the external TMC and Emory datasets (F1 = 0.87-0.93, κ = 0.75-0.86), highlighting its potential for mitigating domain shift across institutions (Supplementary Results and Table S2).

Given the skew toward inactive disease in the test sets, we also assessed precision–recall performance for the active-versus-inactive task (Figure S4). Average precision was 0.95 (LIMUC), 0.96 (EPC), and 0.94 (TMC), well above each cohort’s active-disease prevalence baseline (as low as 0.18 in the remission-skewed LIMUC set), indicating that discrimination was preserved under class imbalance. At the patient level, metrics differed from the corresponding image-level values (Table 2). This reflects the worst-finding aggregation rule, where each patient is labeled and scored by their most severe image, rather than an independent change in model performance. The image-level estimates, computed across all test images, therefore remain the primary measure of performance. More details in Supplementary Methods.

**Table 2.** Image and patient-level performance of the final model (ResNet50), with 95% confidence intervals, and evaluation level (EL).

Dataset	EL	Sensitivity	Specificity	Accuracy	AUROC	Accuracy	QWK	Within-1
		Binary Remission Classification (UC-Re)				Four-class classification (UC-MES)		
**LIMUC**	Image-level	0.82 (0.75-0.88)	0.98 (0.97-0.99)	0.95 (0.94-0.97)	0.98 (0.97-0.99)	0.78 (0.75-0.81)	0.85 (0.81-0.89)	0.99 (0.99-0.99)
**LIMUC**	Patient-level (86)	0.98 (0.92-1.00)	0.91 (0.81-0.98)	0.94 (0.88-0.99)	0.99 (0.97-1.00)	0.74 (0.65-0.84)	0.87 (0.81-0.92)	1.00 (1.00-1.00)
**EPC**	Image-level	0.92 (0.84-0.98)	0.93 (0.86-0.99)	0.93 (0.88-0.97)	0.98 (0.95-0.99)	0.63 (0.53-0.72)	0.84 (0.77-0.88)	0.98 (0.95-1.00)
**EPC**	Patient-level (80)	0.95 (0.86-1.00)	0.93 (0.85-1.00)	0.94 (0.88-0.99)	0.97 (0.94-1.00)	0.59 (0.48-0.69)	0.80 (0.71-0.86)	0.98 (0.94-1.00)
**TMC**	Image-level	0.92 (0.91-0.93)	0.85 (0.84-0.86)	0.88 (0.87-0.88)	0.95(0.94-0.96)	0.61 (0.60-0.62)	0.81 (0.80-0.82)	0.93 (0.93-0.94)
**TMC**	Patient-level	Not applicable, no patient identifiers in the public TMC release						

### 3.3. Grading endoscopic severity (UC-MES model)

The UC-MES model was evaluated for four-class classification of MES 0-3 across the three datasets. Using ResNet50, the model achieved an F1-score of 0.71, an accuracy of 0.78, and a QWK of 0.85 on the LIMUC test dataset (*N* = 1686). The model’s performance on other datasets, along with a comparison with competing architectures, is presented in Table 1. Across all datasets, ResNet50 achieved the highest or near-highest QWK values relative to other architectures, indicating stable ordinal consistency.

Analysis of the confusion matrices (Figure S2C) indicated correct classification rates of 78% for LIMUC, 61% for TMC, and 63% for EPC. Errors were primarily observed between adjacent MES categories. In the LIMUC dataset, out of 370 total misclassifications, 362 (97.8%) were adjacent and eight (2.2%) were non-adjacent. In the TMC dataset, of 3137 misclassifications, 2603 (82.9%) were adjacent and 534 (17.1%) were non-adjacent. For the EPC dataset, 40 (95.2%) were adjacent, while two (4.8%) were non-adjacent. The UC-MES model performance for each preprocessing step is shown in the Supplementary Results and Table S3.

### 3.4. Comparison with expert gastroenterologists in pediatric UC

On the EPC dataset, model performance closely matched that of two independent human reviewers. For the UC-Re, accuracy, F1-score, and Cohen’s kappa were comparable between the model and reviewers, with no statistically significant differences (all *P* > .68) (Figure 3C; Table S4A and Figure S3A). For the UC-MES, overall accuracy was lower (0.63-0.66), but QWK remained high (0.83-0.85), again with no significant differences between the model and reviewers (all *P* > .44) (Figure 3D; Table S4B and Figure S3B). Errors in the four-class task occurred primarily between adjacent categories (MES 1 vs 2 and 2 vs 3). Agreement between the two reviewers (Cohen’s kappa = 0.86 for binary remission; QWK = 0.88 for MES) was in line with the model–reviewer concordance.

### 3.5. Clinical interpretability of model predictions

**Figure 4.**
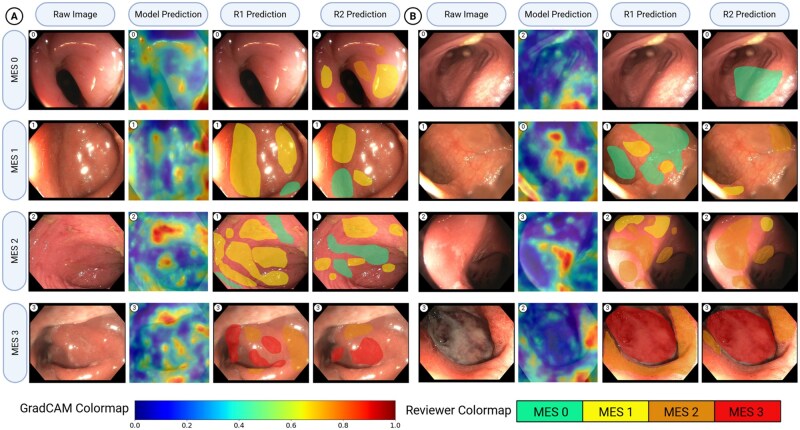
Comparison of ground truth diagnosis with model and reviewer predictions using representative endoscopic images from the EPC cohort. Model predictions are visualized as GradCAM heatmaps; reviewer predictions are displayed as manually annotated pathological zones colored by assigned MES grade (green—MES 0, yellow—MES 1, orange—MES 2, red—MES 3). Reviewers who considered an image MES 0 overall did not annotate a zone. (A) Cases in which the model’s prediction agreed with the ground-truth diagnosis, even when the two human reviewers disagreed, illustrate the model’s capacity for consistent scoring despite inter-observer variability. (B) Cases of model misclassification, in which the model assigned an incorrect MES while at least one human reviewer remained concordant with the ground truth.

Gradient-weighted Class Activation Mapping (Grad-CAM) heatmaps were used to assess the regions contributing to UC-MES model predictions and are shown in Figure 4, with additional cases provided in Figure S5. Across all MES (0-3), heatmaps consistently overlapped with reviewer-annotated pathological zones, demonstrating that the model focussed on clinically relevant mucosal regions. In correctly classified cases, heatmaps showed a graded progression of highlighted areas corresponding to increasing disease severity, from minimal activation in MES 0 to broad, high-intensity regions in MES 3.

Figure 4 further illustrates two distinct prediction scenarios. In Panel A, the model agreed with the ground-truth diagnosis even when the two human reviewers disagreed, demonstrating the model’s capacity for consistent, reproducible scoring under inter-observer variability. In Panel B, the model produced an incorrect prediction, while both human reviewers agreed with the ground-truth label. Importantly, these misclassifications were predominantly confined to the MES 1/2 boundary, the most diagnostically challenging transition in MES grading, and the Grad-CAM heatmaps remained localized to mucosal abnormalities rather than irrelevant image regions. This error pattern suggests that model misclassifications reflect genuine diagnostic difficulty at adjacent grade boundaries rather than systematic feature misattribution.

Taken together, these findings support the model’s clinical interpretability and highlight the MES 1/2 transition as the primary target for future refinement. Additional interpretability analyses are provided in the Supplementary Results.

## 4. Discussion

In this study, we developed and validated a novel, automated AI-based model for MES prediction to address heterogeneity across adult and pediatric datasets from different geographic regions. The study was designed to address three barriers that have limited the clinical translation of AI tools for endoscopic assessment of UC, including methodological heterogeneity, insufficient external validation, and limited cross-institutional generalizability.^31^^,^^32^ By integrating two key technical innovations, FSIH and an optimized ResNet50 architecture, the framework showed encouraging performance across heterogeneous datasets from Asia, the Middle East, and North America, with performance comparable to that of expert gastroenterologists; this direct comparison was limited to the pediatric cohort. These findings suggest that AI-based endoscopic assessment may help improve the consistency and reproducibility of UC disease activity evaluation across different clinical and institutional settings.

Accurate and reproducible endoscopic evaluation is central to UC management, yet substantial inter- and intra-observer variability limits the reliability of human scoring in both clinical practice and trials.^7^^,^^33^ In this context, the UC-Re model addresses a clinically actionable task by distinguishing endoscopic remission (MES ≤ 1) from active disease (MES ≥ 2), a threshold that aligns with treat-to-target strategies and commonly used clinical trial endpoints.^34^ Compared with published benchmarks, our models showed competitive remission-classification performance, including results comparable to DenseNet-based approaches on LIMUC and CB-HRNet-based approaches on TMC.^20^ Importantly, the framework maintained strong performance in two independent external validation cohorts, TMC and EPC, supporting its potential generalizability beyond the development dataset across differences in disease activity distribution and patient characteristics.

Although the UC-MES model showed competitive four-class grading performance compared with larger foundation models such as EndoDINO,^35^ exact four-class MES prediction remained more challenging than binary remission classification. Most misclassifications occurred between adjacent MES categories, particularly at the MES 1-2 boundary, where subtle mucosal differences can lead to disagreement even among experienced readers. Thus, the framework may be most useful in the near term for remission classification, decision support, and flagging borderline cases for expert review, rather than as a stand-alone tool for exact four-class MES grading. More broadly, these two outputs play complementary roles: UC-Re maps onto the guideline-aligned remission (MES 0-1) versus active (MES ≥ 2) target and provides the clinically actionable binary decision, whereas the consistently high QWK and within-1 accuracy of UC-MES indicate that it captures the ordinal structure of disease severity and could support ordinal severity (trajectory) monitoring over time, pending longitudinal validation. Future work should focus on improving MES 1-2 discrimination using larger adjudicated datasets, video-level information, and clinically enriched training cohorts.

Inter-institutional variability is an important barrier to the generalizability of endoscopic AI models. Differences in endoscope systems, image quality, illumination, bowel preparation, mucosal visibility, and local image acquisition practices can introduce substantial domain shift across datasets. In our analysis, the preprocessing pipeline, particularly FSIH, improved external validation performance, supporting its role in reducing site-specific imaging bias. This is important because many prior endoscopic AI models have been evaluated primarily within single-institution or training-adjacent datasets, limiting evidence for external generalizability. These findings suggest that image harmonization may be a useful strategy for improving the transportability of AI-based MES assessment across institutions.

This study extends prior adult-focused AI work by evaluating an adult-developed framework in a pediatric UC cohort. Most AI-based endoscopic diagnostic studies in UC have focused primarily on adults, with limited evaluation in pediatric populations.^11^^,^^13^^,^^18^^,^^19^ The encouraging performance observed in pediatric UC images suggests that adult-trained models may have potential across-age transferability, which could be useful when pediatric training data are limited. However, given the relatively small sample size of 113 images, this transferability requires confirmation in larger pediatric cohorts.

Beyond accuracy, interpretability is essential for clinical adoption of AI systems.^16^^,^^17^ The presented models consistently focussed on diagnostically meaningful mucosal regions while ignoring irrelevant artifacts such as glare, specular reflections, and debris. Grad-CAM visualizations, which closely aligned with reviewer-annotated MES zones, provided transparent insight into model reasoning and enabled clinical auditing of predictions. This interpretability fosters clinician trust and demonstrates that the models base their predictions on physiologically plausible regions, a prerequisite for real-world deployment.^36–38^

Recent studies in this area have begun to evaluate AI-based endoscopic assessment of UC in clinically relevant workflows. Ogata et al.^39^ evaluated AI-assisted video colonoscopy for UC monitoring in a prospective setting and demonstrated its potential for relapse risk stratification and improvement of diagnostic performance among non-specialists. Compared with that work, our study focussed on multicohort evaluation of a static-image framework across adult and pediatric datasets from diverse populations. These approaches are complementary: prospective video-based studies address real-time clinical integration, whereas our study provides evidence regarding cross-cohort transferability across heterogeneous datasets.

The newly presented framework could support several clinical and research workflows. Following prospective validation, the tool could serve as a second-reader tool during post-procedure reporting or image review, allowing clinicians to compare AI-generated MES predictions with their own assessments. The framework may also help standardize longitudinal disease-activity assessment for patients undergoing follow-up colonoscopies across different providers or institutions. In multicenter clinical trials, automated MES prediction could reduce site-to-site variability, support more consistent endpoint assessment, and decrease the burden of central adjudication.

Several limitations warrant consideration. First, the pediatric validation dataset was relatively small; therefore, pediatric performance should be interpreted as preliminary external validation rather than definitive evidence of generalizability. Larger pediatric cohorts would help further evaluate performance across diverse pediatric populations. The cohort was drawn from a single center and the CIs around its pediatric performance estimates are correspondingly wide, so it may not capture the full spectrum of pediatric UC across age subgroups, disease extent, and treatment exposure; prospective multi-center validation is therefore required before any clinical claim of pediatric utility can be supported. Second, ground-truth labeling varied across datasets, with no uniform standard for adjudication. This labeling heterogeneity may have introduced label noise and affected apparent model performance across datasets. Standardized multi-reader adjudication in future studies would help more precisely evaluate model performance across populations and institutions. Third, detailed demographic and clinical metadata were not uniformly available across datasets, limiting assessment of generalizability across patient subgroups. Fourth, while Grad-CAM visualizations generally aligned with pathologic regions, attention to irrelevant areas in a subset of cases highlights the interpretability limitations of gradient-based saliency methods and warrants further investigation. Fifth, our tool was evaluated retrospectively using static endoscopic images; prospective video-based validation will be needed to determine real-time feasibility and clinical utility. Finally, integration of complementary biomarkers, clinical indices, and histopathology may further improve the prediction of therapeutic response and long-term disease course.

This study presents a DL framework for endoscopic assessment of UC that bridges both adult and pediatric populations and demonstrates consistent performance across geographically and institutionally diverse datasets. By incorporating FSIH, the framework showed encouraging performance across heterogeneous evaluation cohorts, supporting its potential to reduce observer- and site-dependent variability in MES assessment. These findings suggest a potential role for AI-assisted endoscopic evaluation as a decision-support tool, although larger adjudicated cohorts and prospective, multicenter, video-based validation are required before routine clinical implementation.

## Supplementary Material

jjag122_Supplementary_Data

## Data Availability

The data underlying this article were obtained under license or permission from Emory University for the Emory pediatric dataset, which was approved under Emory University IRB IRB00115045. Additional data were obtained from publicly available sources, including the TMC-UCM dataset (Tongji Medical College–Ulcerative Colitis with Mayo Scores), accessible at: https://pmc.ncbi.nlm.nih.gov/articles/PMC10432877/#cts13542-sec-0019 and the LIMUC dataset (Labeled Images for Ulcerative Colitis), accessible at: https://zenodo.org/records/5827695#.Yi8GJ3pByUk. Data sharing is subject to institutional approvals and can be made available upon reasonable request to the corresponding author. All endoscopic images used in this study were fully de-identified prior to analysis. For the publicly available LIMUC and TMC datasets, de-identification was performed by the original dataset creators as part of their data release process. For the Emory pediatric dataset (EPC), all patient-identifying information, including metadata and any on-screen text overlays, was removed in accordance with our IRB protocol (IRB00115045) prior to inclusion in this study.
