# Supplementary material for: Automated AI-based Mayo Endoscopic Scoring for ulcerative colitis across adult and pediatric cohorts from diverse populations
Source: J Crohns Colitis. 2026 Aug 18;20(8):jjag122. doi: 10.1093/ecco-jcc/jjag122 (PMC13484516; doi:10.1093/ecco-jcc/jjag122)
Supplement: Supplementary file 1 [file jjag122_supplementary_data.docx]

**Automated AI-Based Mayo Endoscopic Scoring for Ulcerative Colitis Across Adult and Pediatric Cohorts from Diverse Populations**

**Supplementary Methods**

# A. Artifact Detection and Mask Generation

To address the challenge of endoscopic artifacts, we developed an automated detection system based on multi-channel feature analysis. The algorithm processes input images through three parallel transformations: extraction of the green channel (G) from RGB (Red, Green, Blue) space to enhance vascular contrast, saturation (S) from HSV (Hue, Saturation, Value) space to identify low-saturation glare regions, and luminance (L) from LAB (Lightness, A, B) space to detect high-intensity artifacts. These normalized components are combined through the operation F = (1 - S)·G·L, which specifically amplifies regions exhibiting the characteristic low saturation and high luminance of specular reflections. The resulting feature map undergoes cubic scaling (F³) to improve contrast between artifacts and tissue, followed by application of a 3×3 standard deviation filter to suppress normal mucosal texture while preserving artifact boundaries. A fixed-intensity threshold of 0.1 converts the processed map into a binary mask, which is subsequently refined using morphological operations, including hole filling and disk-shaped dilation (radius = 1 pixel), to generate the final artifact mask. This approach demonstrated robust performance across diverse endoscopic imaging conditions, reliably localizing glare-affected areas that could impair diagnostic interpretation if left uncorrected^1^, see Figure S1A.

# B. Contextual Artifact Inpainting (CAI)

For anatomical reconstruction of artifact-obscured regions, we implemented an exemplar-based inpainting algorithm specifically optimized for endoscopic imaging. The system employs tensor-guided patch matching with a fixed patch size of 10 pixels, intelligently propagating surrounding mucosal patterns into artifact regions while maintaining the structural continuity of critical features. This method preserves diagnostically essential elements, including vascular network architecture, subtle inflammatory changes, and ulcer margins, during reconstruction. Validation through blinded side-by-side evaluation by expert endoscopists confirmed that the inpainted images successfully removed artifacts while retaining essential diagnostic features, with no significant distortion of pathological findings. The algorithm's design prioritizes anatomical plausibility over simple texture synthesis, ensuring that reconstructed regions maintain clinical relevance for accurate disease assessment. This approach proved particularly effective for restoring continuity of fine mucosal details that are essential for precise Mayo Endoscopic Scoring ^1^.

# C. Large Mask Inpainting with Fourier Convolutions

Following artifact localization, many endoscopic images in our datasets exhibited common visual disturbances, such as specular glare and instrument shadows. To reconstruct the underlying tissue in these occluded regions, we applied the Large Mask Inpainting with Fourier Convolutions (LaMa) image inpainting strategy. It is a state-of-the-art deep learning (DL) model trained on 4.5 million images, explicitly designed to handle large, irregular masks while preserving both local texture and global structural consistency^2^.

Architecturally, LaMa is a single-stage encoder-decoder network that replaces conventional residual blocks with Fast Fourier Convolutions (FFCs), enabling it to capture both spatial and frequency-domain information. The model processes image-wide context through dual branches: one using traditional convolutions for local features and the other leveraging Fourier transforms to capture long-range dependencies. This structure addresses the limitations of conventional neural networks (CNNs) in handling distant spatial relationships and recurring patterns, see Figure S1A.

During training, LaMa was optimized using a robust loss formulation combining: (1) a patch-level adversarial loss for detailed realism, (2) a high-receptive field perceptual loss to retain semantic consistency, and (3) a feature-matching term that ensures texture and structural alignment across scales. Its aggressive mask-generation strategy, involving wide, polygonal, and arbitrarily shaped regions, trained the network to generalize across diverse occlusions. LaMa’s familiarity with variable image resolutions and its ability to generate anatomically plausible reconstructions make it particularly well-suited for endoscopic imaging, where artifacts often obscure large and non-uniform areas. In this study, we employed the publicly available pre-trained LaMa model without any additional fine-tuning. Prior work has demonstrated its effectiveness for artifact removal in endoscopic imaging tasks^3^.

# D. Fourier-Spatial Image Harmonization

Second, we implemented a two-stage normalization strategy, FSIH, to address domain discrepancies across datasets. This technique leverages histogram matching in both the frequency and spatial domains to align image characteristics while preserving anatomical content, see Figure S1B.  In the first stage, Fourier-domain histogram matching is performed across the entire magnitude spectrum of the image. Unlike earlier approaches, such as Fourier Domain Adaptation (FDA) by Yang and Soatto^4,5^ .Our method aligns the full spectral distribution, which primarily transfers low-frequency components from a target image to a source image. This overcomes a key limitation of the FDA, which may neglect critical variations in mid- and high-frequency bands. Similarly, while Fourier Moment Matching (FMM)^4,5^. aligns only the first and second-order spectral moments; it does not ensure full distributional congruence. In contrast, our method enforces a comprehensive match across all frequency components, improving robustness to complex domain shifts.

The second stage applies spatial histogram matching to the pixel intensity distributions. This refines local details and addresses remaining discrepancies not addressed in the frequency domain, ensuring the final image closely matches the statistical characteristics of the target domain. By combining global frequency alignment with local spatial normalization, FSIH offers a more complete domain harmonization approach. This is particularly important in multi-institutional studies where variations in imaging equipment, protocols, or lighting conditions can introduce subtle yet impactful differences in image appearance. FSIH ensures consistent visual features across datasets, enhancing the generalizability and reliability of downstream DL models.

# E. Deep Learning Model Implementation

This study used Python and MATLAB for model development and data analysis. Deep learning models were implemented using PyTorch and trained on NVIDIA RTX A5500 GPUs. We initialized the ResNet50 architecture with ImageNet-pretrained weights and fine-tuned the network on our endoscopic imaging dataset ^6^. To optimize model performance, we conducted an extensive hyperparameter search evaluating batch sizes (16 vs 32), learning rates (1e-5 to 1e-3), and optimization approaches (stochastic gradient descent [SGD] and Sharpness-Aware Minimization [SAM]) ^7,8^. We selected a final configuration based on validation performance with a batch size of 32, a learning rate of 0.001, a momentum of 0.9, and a weight decay of 0.0001. Multiple established CNN backbones previously used in the literature were also included to compare performance across different tasks.

To explore model decision-making and identify potential failure modes, we applied Gradient-weighted Class Activation Mapping (Grad-CAM) to generate heatmaps highlighting the image regions most contributing to model predictions across various disease severities. For a subset of EPC images, gastroenterologists manually segmented regions they considered diagnostically relevant. Grad-CAM outputs were qualitatively compared with these expert annotations to assess concordance and to examine cases where both clinicians and the model disagreed. This analysis aimed to determine whether model misclassification aligned with regions of diagnostic uncertainty among human reviewers.

In addition to the image-level analysis, model performance was also evaluated at the patient level by aggregating each patient's image predictions into a single prediction. We compared three aggregation strategies: within-patient macro-averaging, mean-probability fusion, and max-probability fusion. Mean-probability fusion was sensitive to within-patient class imbalance: in patients with many remission images and few active images, averaging diluted the active-class probability below the 0.5 threshold, lowering sensitivity. Max-probability fusion was therefore adopted for the binary UC-Re task (patient probability of active disease taken as the maximum image-level probability, classified at the 0.5 threshold), as it matches the clinical convention that any image showing active disease should flag the patient as active; correspondingly, a patient was labeled active in the reference standard if any image was active. For the four-class UC-MES task, the patient-level grade was obtained via expected-grade fusion (i.e., the rounded maximum across each patient's per-image expected MES grade). All 95% confidence intervals were estimated using patient-clustered bootstrap resampling (2,000 resamples)^9,10^, in which patients, rather than individual images, were resampled so that all images from a resampled patient were included together, preserving the within-patient correlation structure and yielding appropriately wide intervals for the smaller external cohorts. Patient-level evaluation was performed for LIMUC (n = 86) and EPC (n = 80); it was not possible for TMC, whose public release contains no patient identifiers.

**Supplementary Results**

## 1. Detection of Remission vs Active Disease

The study evaluated the performance of the UC-Re deep learning model for binary classification of ulcerative colitis remission (Mayo 0–1) versus active disease (Mayo 2–3) across three datasets: LIMUC, TMC, and Emory using different preprocessing methods: raw images (Raw), artificial inpainting models (AIM), and Fourier-Spatial Image Harmonization (FSIH). Results demonstrated that AIM preprocessing yielded the highest F1 score (0.92), accuracy (0.95), and Cohen’s kappa (0.84) on the LIMUC dataset, suggesting strong performance in a controlled setting. In contrast, FSIH preprocessing exhibited superior generalizability, achieving the highest F1 scores (0.93 and 0.87) and Cohen’s kappa values (0.86 and 0.75) on the external TMC and Emory datasets, respectively, see Table S*2*. ROC curves and confusion matrices (Figure S2) confirmed the model’s robustness, which was further supported by an average AUC of 0.966 across all datasets. These findings highlight FSIH’s effectiveness in mitigating domain shift, making it a promising preprocessing approach for cross-institutional applications. However, the smaller sample size of the Emory dataset (N=113) warrants cautious interpretation. Overall, UC-Re, particularly with FSIH preprocessing, shows strong potential for standardized remission classification in diverse clinical settings.

A. **Grading Endoscopic Severity (UC-MES Model)**

The UC-MES model was developed to classify the full spectrum of Mayo Endoscopic Scores (MES 0–3), addressing the increased complexity of distinguishing four disease activity levels. Performance metrics mirrored trends observed in the binary remission task, with AIM preprocessing achieving the strongest internal validation results on the LIMUC dataset (F1 = 0.71, QWK = 0.85), see Table *S3*. However, FSIH demonstrated superior cross-institutional generalization, attaining the highest quadratic weighted kappa (QWK) on the TMC dataset (0.84) and competitive performance on Emory (QWK = 0.81), where raw images showed marginal advantages in accuracy. These findings underscore two key insights: (1) artifact removal (e.g., via AIM) enhances single-institution performance by improving image quality, while (2) FSIH's domain harmonization is pivotal for transferability across heterogeneous datasets. The slight performance dip compared to the binary task reflects the inherent challenge of fine-grained classification, yet the model maintained robust discrimination even in this four-class setting, supporting its potential for comprehensive endoscopic severity assessment.

## B. Model Interpretability and Clinical Validation

Grad-CAM visualizations (Figure S5) demonstrate that both the UC-Re and UC-MES models base their predictions on clinically relevant endoscopic features, mirroring human expert assessment. Each Mayo score exhibits distinct pathological characteristics: Mayo 0 (normal) shows minimal, diffuse activation correctly indicating the absence of focal pathology; Mayo 1 (mild) reveals localized attention to early disease signs such as erythema and subtle vascular changes; Mayo 2 (moderate) shows stronger activation at ulcer margins and distorted vasculature; and Mayo 3 (severe) displays intense, confluent attention over deep ulcers with complete vascular pattern loss. Notably, the models consistently disregard non-pathological artifacts such as specular reflections and debris, focusing exclusively on diagnostically significant features. This clinical alignment is particularly valuable in the challenging Mayo 1–2 boundary, where the model's attention to transitional vascular abnormalities may explain its superior performance relative to human reviewers. In pediatric cases especially, where detecting subtle mucosal changes critically impacts treatment decisions, such transparent decision-making builds the trust necessary for AI-assisted endoscopy. By validating both the decision process and the biological plausibility of predictions, these visualizations effectively bridge artificial intelligence with clinical practice, supporting integration into real-world diagnostic workflows.

**Supplementary Figures**

Figure S1. Overview of the preprocessing pipeline. (A) Illustration of the LaMa (Large Mask Inpainting) process. From left to right: the original endoscopic image, detection of artifact regions (highlighted in green), schematic of the LaMa architecture with Fast Fourier Convolution (FFC) residual blocks, and the resulting artifact-corrected image. This step removes specular glare and restores mucosal detail through context-aware inpainting. (B) Schematic of the Fourier-Spatial Image Harmonization (FSIH) workflow that contains two steps: (1) Fourier histogram matching and (2) spatial histogram matching.

Figure S2. Confusion matrices for the UC-Re and UC-MES models across three independent datasets (LIMUC, TMC, and Emory pediatric). (A) Binary UC-Re task (remission classification, MES 0–1 vs. 2–3), showing strong discrimination across all datasets (average AUC 0.97). (B) Extreme-case contrast (MES 0 vs. 3), showing near-perfect separation between complete remission and severe disease (average AUC 0.999). (C) Four-class UC-MES task (MES 0, 1, 2, 3), showing high classification accuracy across all datasets.

Figure S3. Confusion matrices comparing the model with independent human reviewers on the Emory pediatric cohort (EPC). R1 and R2 are two independent board-certified gastroenterologists, both blinded to the original ground-truth diagnosis. (A) Binary UC-Re task (remission classification, MES 0–1 vs. 2–3): model predictions against ground-truth (left) and R1 against R2 (right). (B) Four-class UC-MES task (MES 0–3): model predictions against ground-truth (left) and R1 against R2 (right). Agreement between the two reviewers was comparable to the model's agreement with the ground truth across both tasks. UC-Re, UC remission classification; UC-MES, Mayo Endoscopic Subscore grading; EPC, Emory pediatric cohort.

Figure S4. Precision–recall curves for the active (MES ≥2) versus inactive (MES 0–1) classification on each test set. Average precision (area under the PR curve) is 0.952 (LIMUC), 0.964 (EPC), and 0.941 (TMC). Dotted lines indicate each cohort's active-disease prevalence (the random-classifier baseline). High AP well above baseline, including on the remission-skewed LIMUC set (baseline 0.18), indicates that discrimination is maintained under class imbalance.

Figure S5. Grad-CAM visualizations highlighting discriminative regions used by the DL model to classify endoscopic images across the four Mayo endoscopic score categories (0–3) from the TMC cohort. The top row displays representative endoscopic images, while the bottom row shows corresponding Grad-CAM heatmaps, where red denotes regions with the highest influence on the model’s prediction.

**Supplementary Tables**

| *Table S1. Distribution of the labels across three datasets (LIMUC, TMC, and EPC).* | | | | | | | |
| --- | --- | --- | --- | --- | --- | --- | --- |
| **Cohort Name** | **Average Age (years)** | **Male: Female** | **Mayo 0** | **Mayo 1** | **Mayo 2** | **Mayo 3** | **Total** |
| **LIMUC** | 43.3 +- 13.7 | 56.5%:43.5% | 6105 (54.14%) | 3052 (27.70%) | 1254 (11.12%) | 865 (7.67%) | 11276 |
| **TMC** | NA | NA | 2996 (37.55%) | 1800 (22.56%) | 1721 (21.57%) | 1461 (18.31%) | 7978 |
| **EPC** | 11 | 51%:49% | 41 (36.28%) | 20 (17.70%) | 29 (25.66%) | 23 (20.35%) | 113 |

| *Table S2. Performance metrics for remission classification across three datasets (LIMUC, TMC, and EPC) using raw images (Raw), artificial inpainting models (AIM)-based inpainting, and Fourier-Spatial Image Harmonization (FSIH).* | | | | | | | | | | | | | | | |
| --- | --- | --- | --- | --- | --- | --- | --- | --- | --- | --- | --- | --- | --- | --- | --- |
|  | **LIMUC (N=1686)** | | | | | **TMC (N=7978)** | | | | | **Emory (N=113)** | | | | |
|  | **Pre** | **Rec** | **F1** | **ACC** | **Kap** | **Pre** | **Rec** | **F1** | **ACC** | **Kap** | **Pre** | **Rec** | **F1** | **ACC** | **Kap** |
| **Raw** | 0.94 | 0.87 | 0.90 | 0.94 | 0.81 | 0.87 | 0.88 | 0.87 | 0.87 | 0.74 | 0.63 | 0.59 | 0.66 | 0.66 | 0.28 |
| **AIM** | 0.93 | 0.90 | 0.92 | 0.95 | 0.84 | 0.86 | 0.87 | 0.85 | 0.86 | 0.72 | 0.89 | 0.90 | 0.90 | 0.91 | 0.80 |
| **FSIH** | 0.93 | 0.87 | 0.90 | 0.95 | 0.80 | 0.87 | 0.88 | 0.87 | 0.88 | 0.75 | 0.93 | 0.93 | 0.93 | 0.93 | 0.86 |

| *Table S3. Performance metrics for Mayo Endoscopic Score (MES) classification across three datasets (LIMUC, TMC, and Emory) using raw images (RImg), artificial inpainting models (AIM)-based inpainting, and Fourier-Spatial Image Harmonization (FSIH).* | | | | | | | | | | | | | | | |
| --- | --- | --- | --- | --- | --- | --- | --- | --- | --- | --- | --- | --- | --- | --- | --- |
|  | **LIMUC (N=1686)** | | | | | **TMC (N=7978)** | | | | | **Emory (N=113)** | | | | |
|  | **Pre** | **Rec** | **F1** | **ACC** | **QW** | **Pre** | **Rec** | **F1** | **ACC** | **QW** | **Pre** | **Rec** | **F1** | **ACC** | **QW** |
| **Raw** | 0.73 | 0.68 | 0.70 | 0.77 | 0.84 | 0.67 | 0.66 | 0.64 | 0.69 | 0.86 | 0.65 | 0.53 | 0.51 | 0.55 | 0.69 |
| **AIM** | 0.72 | 0.71 | 0.71 | 0.78 | 0.85 | 0.66 | 0.65 | 0.62 | 0.68 | 0.85 | 0.72 | 0.64 | 0.62 | 0.62 | 0.80 |
| **FSIH** | 0.66 | 0.66 | 0.66 | 0.74 | 0.83 | 0.57 | 0.56 | 0.49 | 0.61 | 0.81 | 0.63 | 0.64 | 0.62 | 0.63 | 0.84 |

References:

1.	Kudva, V., Prasad, K. & Guruvare, S. Andriod Device-Based Cervical Cancer Screening for Resource-Poor Settings. *J. Digit. Imaging* **31**, 646–654 (2018).

2.	Suvorov, R. and L. E. and M. A. and R. A. and A. A. and S. A. and K. N. and G. H. and P. K. and L. V. Resolution-robust large mask inpainting with fourier convolutions. *Proceedings of the IEEE/CVF winter conference on applications of computer vision* 2149–2159 (2022).

3.	Li, W., Jia, F. & Liu, W. EndoSRR: a comprehensive multi-stage approach for endoscopic specular reflection removal. *Int. J. Comput. Assist. Radiol. Surg.* **19**, 1203–1211 (2024).

4.	Yang, Y. & Soatto, S. Fda: Fourier domain adaptation for semantic segmentation. in *Proceedings of the IEEE/CVF conference on computer vision and pattern recognition* 4085–4095 (2020).

5.	Krishnamachari, K., Ng, S.-K. & Foo, C.-S. Mitigating real-world distribution shifts in the fourier domain. *Transactions on Machine Learning Research* (2023).

6.	Deng, J. *et al.* ImageNet: A large-scale hierarchical image database. in *2009 IEEE Conference on Computer Vision and Pattern Recognition* 248–255 (IEEE, 2009). doi:10.1109/CVPR.2009.5206848.

7.	He, K., Zhang, X., Ren, S. & Sun, J. Deep Residual Learning for Image Recognition. in *2016 IEEE Conference on Computer Vision and Pattern Recognition (CVPR)* 770–778 (IEEE, 2016). doi:10.1109/CVPR.2016.90.

8.	Paszke, A. Pytorch: An imperative style, high-performance deep learning library. *arXiv preprint arXiv:1912.01703* (2019).

9.	Efron, B. & Tibshirani, R. J. *An Introduction to the Bootstrap*. (Chapman and Hall/CRC, 1994). doi:10.1201/9780429246593.

10.	Field, C. A. & Welsh, A. H. Bootstrapping Clustered Data. *J. R. Stat. Soc. Series B Stat. Methodol.* **69**, 369–390 (2007).
